# DAILY HEALTH PROCESSES IN POPULATIONS OF DIVERSE DEMOGRAPHIC BACKGROUNDS

**DOI:** 10.1093/geroni/igae098.1883

**Published:** 2024-12-31

**Authors:** Christina Roecke, Minxia Luo, Allison Bielak

**Affiliations:** Chair; Co-Chair; Discussant

## Abstract

Health processes are studied increasingly commonly in older adults’ daily life. This symposium includes four studies that investigated daily health processes in populations of diverse demographic backgrounds and explored differences in the daily processes between individuals. First, with seven-day data from 207 adults aged 18-82 years, Ferguson, Corley, and Scott found that negative affect was associated with occurrence of stressors at both the within-person momentary and between-person levels, but age or neuroticism did not moderate the association. Second, Ng, Kratz, and Birditt conducted a five-day study on effects of interactions with care recipients and with friends on cardiovascular functioning in 159 Black and White dementia caregivers. In particular, they found that negative interactions with care recipients were linked to momentary increases in HR in Black but not in White caregivers. Third, Luo, Röcke, and colleagues investigated the short-term time-lagged associations between leisure activities and cognitive performance over hours and the differences across age, gender, education, and ethnicity. They analyzed data from two two-week studies from a total 542 young and older adults with diverse ethnicity backgrounds, but did not find any time-lagged associations. Fourth, Hartung and Hülür examined daily subjective memory lapses in relation to cognitive activity and mood in 325 university students over 10 days. Their results showed that memory lapses were associated with more cognitive activity and worse mood both momentarily and between individuals. Finally, Allison Bielak will discuss all papers about generalizability of observations of daily health processes across populations of different demographic backgrounds.

